# A cross-sectional survey to investigate the quality of care in Tuscan (Italy) nursing homes: the structural, process and outcome indicators of nutritional care

**DOI:** 10.1186/s12913-015-0881-5

**Published:** 2015-06-06

**Authors:** Guglielmo Bonaccorsi, Francesca Collini, Mariangela Castagnoli, Mauro Di Bari, Maria Chiara Cavallini, Nicoletta Zaffarana, Pasquale Pepe, Alessandro Mugelli, Ersilia Lucenteforte, Alfredo Vannacci, Chiara Lorini

**Affiliations:** Department of Health Science, University of Florence, viale GB Morgagni 48, 50134 Florence, Italy; Regional Health Agency of Tuscany, Tuscany, Italy; Research Unit of Medicine of Aging, Department of Experimental and Clinical Medicine, University of Florence-Unit of Geriatric Cardiology and Medicine, Florence, Italy; Department of Heart and Vessels, Azienda Ospedaliero-Universitaria Careggi, Florence, Italy; Department of Neuroscience, Psychology, Drug Research and Children’s Health, University of Florence, viale Pieraccini 6, 50139 Florence, Italy

**Keywords:** Quality indicators, Nutritional care, Nursing home, Multilevel analysis, Case mix, Risk factors

## Abstract

**Background:**

Previous studies have investigated process and structure indicators of nutritional care as well as their use in nursing homes (NHs), but the relative weight of these indicators in predicting the risk of malnutrition remains unclear.

Aims of the present study are to describe the quality indicators of nutritional care in older residents in a sample of NHs in Tuscany, Italy, and to evaluate the predictors of protein-energy malnutrition risk.

**Methods:**

A cross-sectional survey was conducted in 67 NHs. Information was collected to evaluate quality indicators of nutritional care and the individual risk factors for malnutrition, which was assessed using the Malnutrition Universal Screening Tool. A multilevel model was used to analyse the association between risk and predictors.

**Results:**

Out of 2395 participants, 23.7 % were at high, 11 % at medium, and 65.3 % at low risk for malnutrition. Forty-two percent of the NHs had only a personal scale to weigh residents; 88 % did not routinely use a screening test/tool for malnutrition; 60 % used some standardized approach for weight measurement; 43 % did not assess the severity of dysphagia; 12 % were not staffed with dietitians.

Patients living in NHs where a chair or platform scale was available had a significantly lower risk of malnutrition (OR = 0.73; 95 % CI = 0.56–0.94). None of the other structural or process quality indicators showed a statistically significant association with malnutrition risk.

**Conclusions:**

Of all the process and structural indicators considered, only the absence of an adequate scale to weigh residents predicted the risk of malnutrition, after adjusting for case mix. These findings prompt the conduction of further investigations on the effectiveness of structural and process indicators that are used to describe quality of nutritional care in NHs.

## Background

As a combined effect of population aging, of the increase in the number of older persons with disabilities related to multiple co-morbidities, and of changes in family structure, in recent decades nursing homes (NHs) have being playing an increasing role in the care of elderly persons.

Quality of care is a multidimensional construct that is *per se* difficult to define and assess in NHs [1, 2]. Specifically, only recently this concept and its dimensions of effectiveness, efficiency, safety, equity, and appropriateness have been developed and applied to Italian NHs, whereas they are commonly considered for hospitals. A few recent studies have been conducted to describe and better understand the kind of services provided, at national and regional levels, to residents of Italian NHs with different levels and types of disabilities, and to assess the quality of these services [3–5]. Furthermore, both the central government and regional authorities are taking actions and developing specific legislations to ensure quality of care and equity of access to older persons with disabilities and NHs residents across the whole nation [6–8].

The scientific evidence on which tools and practices are most effective in maintaining NH residents’ abilities is scarce and, therefore, it is still unclear which measures should be included in quality indicators. However, it may be argued that, similar to other settings, quality indicators of structure, process, and outcomes should be used in NHs [2]. Among other aspects, the assessment of the risk for protein-energy malnutrition deserves special attention. Malnutrition has a strong impact on the quality of life of older NH residents, increases mortality, morbidity, and length of hospital stay, delays recovery after acute events, reduces autonomy, worsens the outcome of many clinical events, and increases health care costs [9]. Yet, assessment of malnutrition risk remains largely unapplied in NHs, despite the high prevalence, up to 85 %, of malnutrition among older persons living in this setting [9].

Some authors have investigated the role of process or structure indicators of nutritional care as well as their use in NHs. The Minimum Data Set (MDS) assessment contains some specific indicators to assess the quality of nutritional care as well [10–12].

In particular, the use of a routine nutritional screening procedure, the availability of dietitians and the use of protocols or guidelines for specific nutritional requirements are the most commonly investigated aspects [13]. However, the relative weight of each structure or process indicator in predicting the outcome indicator (i.e., malnutrition risk) remains unclear. In fact, even if, according to Donabedian’s framework of quality of care [14], the outcome depends on the process of care and on structural aspects of care, only few studies investigated the quantitative relationship between structure or process indicators and outcome indicators of malnutrition [15, 16].

In addition to the aspects related to quality of care, several other factors may influence the risk of malnutrition in older persons, most of which are related to individual resident’s characteristics, such as age, gender, comorbidity, and functional and cognitive impairments. These factors are only partially affected by the quality of care and are important confounders to consider when evaluating the quality of the nutritional care process and the validity and reliability of the quality measures [17, 18].

This study aimed to describe structural, process, and outcome indicators of nutritional care in a sample of NHs in Tuscany, Italy. The study also aimed to evaluate the predictors, either individual or related to the quality of care, of malnutrition risk among the elderly residents and to evaluate the appropriate weighting of the structural and process indicators in predicting the outcome indicators of malnutrition, controlling for individual risk factors.

## Methods

### Setting and study design

The study was designed according to the principles of the Helsinki Declaration and its protocol was approved by the Regional Committee for Bioethics of the Tuscany Region and by the Ethics Committee of the Local Health Units of Siena, Firenze and Pisa. As detailed in a previous publication [19], it has been conducted as a part of the project “Monitoring the quality of care in nursing homes”. Briefly, 67 NHs (approximately 22 % of all Tuscan NHs) volunteered to participate between 2011 and 2012 and enrolled 2801 older residents. Data were collected in a cross-sectional survey; 89 NH staff members were specifically trained by the research team on how to collect the data. To the purpose of the present study, only residents aged 65 + years were considered.

The survey was conducted between January and March 2012. NHs were grouped based on their geographical location; in each area, the survey lasted a month, including a 16-h training session for the staff and the cross-sectional survey (1 week).

The quality indicators considered in the overall survey, reported in Table 1, were chosen upon literature review, as detailed elsewhere [20]. The information needed to obtain the structural, process, and outcome indicators related to protein-energy malnutrition, as well as those related to the individual malnutrition risk factors (which should be considered as confounders for the purpose of this study), were collected using three forms (Table 1): in the first one, information on the NH was recorded, whereas the two others recorded individual nutritional risk factors and other residents’ characteristics, useful to describe the facility case-mix, respectively. Thus, data were collected at two levels, the institution (for structural and process indicators) and the resident one (for the outcome indicator and the case-mix). The resident’s forms included both *ad hoc* items and validated tests or widely used scales, selected and shared within the multidisciplinary research group. Individual characteristics, such as mood, behaviour, communication, hearing and sight problems, were included as parts of multidimensional assessment scales. In particular, a modified version of the Barthel Index was used to measure the subjects’ performance in activities of daily life, where a score of 100 indicates “complete dependency” [34, 35]; the Pfeiffer test was used to assess cognitive deficits [36]; the Eating Behaviour Scale to measure patients’ functional ability during eating [37].

**Table 1:** Quality indicators selected for the study and content of the forms used for collecting data

Quality indicators	Collected information		
	Form for collecting data on nutritional care at NHs level	Form for collecting data on nutritional care at individual level	Form for collecting data on case-mix at individual level
Structural indicators [13]:			
● type of scales used to weigh residents (if any)	● Type of scales used to weigh the residents (if any)	● Personal data (date of birth, gender)	● Length of stay in NH
● employment of dietitians and type of consultation	● Type of nutrition screening tool applied (if any) and frequency of utilisation	● Number of hospital admissions during the 12 months before the day of the survey	● Severe dementia (Global Deterioration Scale) [23]
● number of operators assigned to the administration of meals in a specific day	● Presence and use of protocols or guidelines for weight assessment	● Data used to calculate the MUST score (anthropometric data, unplanned weight loss in previous 3–6 months, whether the resident is acutely ill and whether there has been or is likely to be no nutritional intake for more than 5 days)	● Bedridden for almost 18 h/day
Process indicators [13]:			
● use of a nutrition screening tool	● Presence and use of protocols or guidelines for administration of food	● Feeding mode (enteral feeding, parenteral feeding, syringed-feeding, texture-modified diet)	● Physical impairment (Barthel Index, 100 = “completely dependent”) [24]
● presence of protocols or guidelines for weight assessment	● Assessment of dysphagia	● If the subject is following a program to change his body weight	● Cognitive impairment (Pfeiffer test score) [25]
● presence of protocols or guidelines for administration of food	● Employment of dietitians	● Number of own teeth and presence of dentures or removable bridges	● Mood and behaviour pattern, SOSIA form [26]
● Assessment of dysphagia	● Number of operators assigned to administration of meals in a specific day	● Functional ability during eating, using the Eating Behaviour Scale [21]	● Communication, hearing and vision patterns, SVAMA schedule [27, 28]
		● Semi-quantitative assessment of the amount of food consumed at lunch in the day of the survey, according to similar studies and contexts [22], with indications regarding the intake of complementary or substitute supplements, the causes of the partial consumption of lunch and the place in which the lunch was consumed	
Outcome indicators:			
● Prevalence of subjects with medium-high risk of malnutrition		● Place where meals were consumed on the day of the survey (dining room or bedroom)	● Comorbidity, according to Disease Count (DC) [28]
		● Presence of dysphagia (yes/no)	

Information was collected and recorded by trained staff, using both direct observation and clinical judgement and consulting medical records. Forms were designed for optical reading and were collected anonymously, though uniquely identifiable from individual and NH codes. The director of each NH collected the forms and sent them to the Regional Health Agency of Tuscany for data entry and analysis.

### Detection of anthropometric measurements and assessment of malnutrition risk

The collection of anthropometric measurements and the evaluation of the risk of malnutrition have been described in a previous article [19].

The risk of malnutrition was assessed using the Malnutrition Universal Screening Tool (MUST) [30, 31]. The MUST was developed to assess the nutritional status and the need for nutrition support in adults across all care settings [31, 32]. It is a simple, quick tool with proven excellent reliability between health care workers and fair-good-to-excellent agreement with regards to the detection of malnutrition compared with other tools [20, 31, 33]. The MUST consists of 5 consecutive steps, the first of which is to calculate the Body Mass Index (BMI) from subject’s weight and height, both of which are obtained using standard, direct methods. As an alternative, height can be estimated using measures of some body segments, such as knee height and ulna length. Similarly, the BMI can be estimated from the Middle Upper Arm Circumference (MUAC) when weighing the subject is impossible [34, 39]. The second step of the MUST evaluates the percentage of involuntary weight loss in the previous 3–6 months; the third indicates the presence of an acute disease, that may have prevented the patient from eating within 5 days prior to data collection or suggests that no nutritional intake has occurred for at least 5 days; in the fourth step, the scores obtained in the three previous steps are summed, and the resulting score is used to assign the patient to a low-, medium-, or high-risk category in the following fifth step, where corresponding management guidelines are eventually suggested.

Weight, height, knee height (KH), ulna length (UL) and MUAC were collected, whenever possible, i.e. when a scale and a stadiometer were available in the NH and when the residents agreed to be measured and their health state was compatible with the detection of measurements using standardised methods [40]. All of the measurements were obtained according to the indications reported in the MUST report 39, using the scales and stadiometers that were available in each NH. Each ward was also equipped with an inelastic meter for measuring the body segments and the MUAC. Using the MUST equations, the KH and UL were used to estimate height, while the MUAC was used to estimate the BMI categories (<18.5; 18.5–20; >20 kg/m^2^). The weight and height (measured and/or estimated) were used to calculate the BMI.

As described in a previous study [19], the MUST score was calculated according to the following criteria: 1) using the BMI value obtained with direct measurements, if possible; 2) otherwise, considering alternative anthropometric measurements in a pre-set order: weight and estimated height from ulna length; weight and estimated height from knee height; and MUAC.

In agreement to MUST report [39], residents were classified as at low-, medium-, and high-risk for malnutrition for scores of 0, 1, or >1, respectively.

### Statistical analysis

Data are presented as mean ± standard deviation or as percentages. Bivariate associations were assessed with the *χ*^2^ test, comparisons between means were performed using Student’s *t*-test for independent samples.

To evaluate the predictors of malnutrition risk among the elderly residents and the appropriate weighting of the structural and process indicators in predicting the outcome indicator of malnutrition, the original three MUST categories were collapsed into two classes, grouping together subjects at medium- and high-risk in the dichotomous variable “MUST indicator”. Because of the public health perspective of the research, we decided to separate residents at low- from those at higher risk without stratifying the higher risk categories.

To account for the clustering of patients into each NH, a random intercept logit model was used [41, 42]. Indeed, when the data present some type of hierarchy (i.e. clustering of patients into NH), standard models, such as logistic regression, might not be adequate and can provide misleading results: in particular, standard errors may be underestimated, leading to type I error rates higher than the nominal α level. Therefore, to estimate the proportion of total variability due to the clustering of patients into NH, a random intercept logit model was defined in order to analyse the association between the MUST indicator, taken as an outcome variable of low- vs. medium-high risk category, and possible explanatory variables (covariates).

Considering all the explanatory variables (either the process and structural indicators or individual characteristics), the fixed part of the model (the covariates) was selected based on the Akaike Information Criteria (AIC). A further adjustment for age and gender was also performed. The random part was tested according to the respective *χ*^2^ test. The variables selected and included in the final model were: the Barthel Index score, the Pfeiffer test score, the EBS scale score, age, gender, where the subject consumed lunch on the day of the survey (dining room *vs* bedroom) and the type of available scale (no scale, only personal scale, chair or platform scale), leading to the following final model:

Fixed part: MUST_ij_ = b_0j_ + b_1j_Barthel _ij_ + b_2j_Pfeiffer_ij_ + lunch _ij_ + b_4j_ EBS _ij_ + b_5j_ age _ij_ + b_6j_ gender_ij_ + e_ij_

Random part (intercept): b_0j_ = λ_00_+ λ_01_scale + u_0j_.

No data imputation was performed for missing values; therefore, the final multilevel model was based on the sole 2026 participants with no missing values in all the variables included.

Goodness of fit of the final model was measured from the area under the ROC curve.

The analyses were performed with STATA12 (StataCorp, College Station, Texas 77845 USA); specifically, logit model has been performed by means of xtlogit function. Statistical significance was set at an α level of 0.05.

## Results

### Individual assessments (prevalence of malnutrition risk)

We were able to assess the malnutrition risk for 2395 residents (74.5 % females, 25.2 % males) aged > 64 years, representing 92 % of all subjects enrolled.

The percentage of missing data was:

- 40 % for the variables related to own teeth, dentures and removable bridges;
- between 6 and 15 % for the variables related to mood and behaviour, communication, hearing and sight, and for dysphagia assessment;
- ≤6 % for all the other variables.

A quarter of the sample was aged 90 + years. Females were, on average, significantly older than males (85.6 ± 7.9 vs. 80.5 ± 7.9 years; *p* < 0.001).

Subjects at high-, medium-, and low-risk for malnutrition were 65.3, 11 and 23.7 %, respectively.

The risk of malnutrition was significantly associated with the following variables (Table 2): presence of severe dementia, being bedridden, poor language understanding and expression, impaired visual and hearing abilities, nervousness, physical impairment, cognitive impairment, feeding mode (enteral feeding, parenteral feeding, syringe-feeding texture-modified diet), place where meals were consumed on the day of the survey, age and functional ability related to eating. In particular, the highest prevalence of medium-high risk of malnutrition was observed in oldest subjects, in the presence of higher levels of physical or cognitive impairment or of other conditions associated with very poor health status (i.e., subjects bedridden in enteral or parenteral nutrition).

**Table 2:** Percentage of subjects (*N* = 2395) with medium-high risk of malnutrition by individual variables

Variables (*N*)		% subjects at medium-high risk of malnutrition	*p*
Gender	Males (*611*)	29.7	0.003
	Females (*1784*)	36.4	
Age	65–79 (*641*)	27.8	<0.001
	80–85 (*579*)	32.0	
	86–90 (*622*)	36.2	
	>90 (*553*)	43.8	
Severe dementia	Yes^b^ (*640*)	50.5	<0.001
>18 h/day bedridden	Yes (*205*)	62.4	<0.001
Language understanding	Do not understand or it is not possible to evaluate the level of comprehension (*520*)	55.2	<0.001
Language expression	Understanding only isolated words or not expressed (*579*)	51.1	<0.001
Hearing	Severely impaired or deaf (*166*)	42.8	0.002
Vision	Severely impaired or blind (*169*)	43.8	0.001
Gastrointestinal diseases	Yes (*366*)	36.9	0.310
Respiratory diseases	Yes (*403*)	34.5	0.976
N diseases (DC)	>4 (*830*)	35.1	0.643
Confusion/awareness	Completely disoriented (*994*)	43.1	<0.001
Nervousness	Easily irritable (*516*)	37.8	0.002
Restlessness/calmness	Restless, unable to sit still (*198*)	38.4	<0.001
Physical impairment	Severe^a^ (*1344*)	45.3	<0.001
Cognitive impairment	Severe^c^ (*1089*)	47.3	<0.001
N hospital admissions	1 or more (*490*)	36.5	0.491
Disability during eating	Severe^d^ (*590*)	55.1	<0.001
Enteral nutrition	Yes (*79*)	65.8	<0.001
Syringed feeding	Yes (*44*)	77.3	<0.001
Parenteral nutrition	Yes (*32*)	59.4	0.003
Texture modified diet	Yes (*414*)	54.8	<0.001
Enriched diet	Yes (*87*)	72.4	<0.001
Dysphagia	Yes (*58.1* %)	36.8	0.054
Lunch location	Dining room (*2128*)	31.6	<0.001
	Bedroom (*214*)	59.3	
Teeth	Edentulous without dentures/bridges (*399*)	60.7	0.001
	Edentulous with dentures/bridges (*636*)	39.8	
	1–20 teeth without bridges (*571*)	47.1	
	1–20 teeth with bridges (*338*)	32.8	
	>20 teeth (*201*)	35.8	
Totals		34.7	

^a^Barthel scale score 60–100

^b^Level 7 of the Global Deterioration Scale

^c^Pfeiffer test score > 7

^d^EBS score > 12

### Characteristics of the NHs (structural, process and outcome indicators at the NH level)

**Fig. 1:**
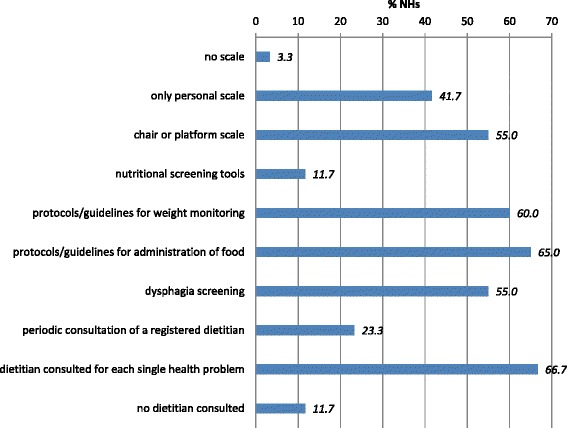
The structural and process quality indicators of nutritional care

NH characteristics with regards to the structural and process indicators of nutritional care are shown in Fig. 1.

Of the NHs surveyed, 3 % did not have any scale to weigh residents, 42 % had only a personal scale and 55 % had also a chair or a platform scale. Additionally, 88 % did not routinely use a malnutrition screening test/tool; when used, the most frequently applied screening tests were the MUST (4 NHs) and the Mini Nutritional Assessment (MNA) (3 NHs). Protocols, procedures or guidelines for weight assessment were used in 60 % of the NHs, and 65 % used such guidelines for the administration of food. While dietitians or nutritionists were present in 88 % of the NHs, they routinely assessed the nutritional status of all residents in only 14 NHs (26.4 % of those where these healthcare professionals were employed), while in the majority of cases (83 %) the intervention was on-demand, only to manage individual health problems (i.e., diets for specific diseases).

The degree of dysphagia was not assessed in 43 % of the NHs.

The prevalence of the medium-high risk of malnutrition ranged between 4.5 and 64.1 % (mean: 34.9 ± 11.2 %, median: 35.8 %).

The median number of residents per operator assigned to the administration of meals in a specific day was 6.3 (range: 2.3–27.5; mean: 7.4 ± 4.4).

### Multilevel model

The results of the random intercept logit model (OR with their 95 % confidence interval) with regards to MUST indicator are reported in Table 3.

**Table 3:** The random-effects logistic regression for MUST indicator

	OR	[95 % CI]
Barthel scale score	1.01	[1.00; 1.01]
Pfeiffer test score	1.07	[1.03;1.11]
Eating in the bedroom	1.58	[1.07; 2.32]
EBS score	0.95	[0.94; 0.97]
Chair or platform scale vs other	0.73	[0.56; 0.94]
Age (years)	1.02	[1.01; 1.04]
Gender (males *vs* females)	0.96	[0.75; 1.23]

Number of observations = 2026, Number of groups = 58, Area Under ROC curve = 0.7091

As regard to the individual characteristics, the variables that maintained a statistically significant association with the outcome were age, measures of physical or cognitive impairment (Barthel Index, Pfeiffer test score, EBS score) and the place where meals were consumed on the day of the survey. For each one unit increase in the Barthel Index, Pfeiffer test score, EBS score and age, the odds of being at medium-high risk of malnutrition were 1.01, 1.07, 0.95, and 1.02, respectively.

Patients who ate in their bedroom had an OR of 1.58 for being at medium-high risk of malnutrition, compared with those who ate in the dining room.

Patients living in NHs where a chair or platform scale was available had significantly lower risk of malnutrition (OR = 0.73), compared to those living in NHs that do not have any kind of scale or have only personal scale. None of the other structural or process quality indicators showed a statistically significant association with the outcome indicator. The proportion of total variability due to the clustering of patients into NH was only 2.6 % (1.0–7.0 %), implying that while patients within each NH may be quite dissimilar, there is not a great difference between the groups. The area under the ROC curve was 0.709, showing the fair accuracy of the model in discriminating between patients at medium-high or low risk of malnutrition.

## Discussion

Our results demonstrate that, overall, NHs in Tuscany obtained suboptimal scorings in structural and process quality indicators for nutritional care. In particular, the presence of dietitians in the NH staff and routine, standardized assessment of malnutrition were unacceptably infrequent (23.3 and 11.7 %, respectively), whereas the frequency of application of protocols and guidelines for weight assessment and administration of food, though greater (60 and 65 %) compared to other indicators, remains lower than previously reported in similar settings [13, 43–45]. Additionally, it is important to highlight that in as many as 43 % of the NHs the degree of dysphagia was not evaluated with standardized tools (43 %), and that scales to weigh residents with severely impaired mobility were unavailable in approximately 60 % of the NHs, whereas a small (3.3 %) of them has no scales whatsoever. These indicators have generally not been reported in other studies, perhaps because they are considered basic requirements and not a measure to be examined.

Despite the infrequent use of protocols, guidelines or tools, the value of the specific outcome indicator adopted in our study (i.e., prevalence of malnutrition risk according to the MUST) is in line with previous reports in the literature [9]. This discrepancy can be explained by the presence of non-coded behaviours that may reduce the risk of malnutrition (i.e. special attention in the selection of meals for individuals who have lost weight, even in the absence of specific protocols). This hypothesis is in agreement with reports by other authors on the effectiveness of structure and process indicators in describing the quality of care [7, 9]. The operational tools available do not always reflect the activities and precautions taken by the staff during the process of care, so it should not be assumed that the more frequent use of structural and process indicators implies better nutritional care or *vice versa*. In the study conducted by Meijers et al. [16] a lower prevalence of malnutrition was associated with more frequent application of nutritional screening and oral nutritional supplementation, but not with other structural or process quality indicators. Instead, in the study performed by van Nie at al. [15], the prevalence of malnutrition was associated with five structural or process indicators.

The results of our regression analysis show that among the process and structural indicators included in our study, the only one with a role in predicting malnutrition risk after adjusting for individual risk factors (reflecting the case-mix) was the availability of a scale suitable to weigh residents even in the presence of mobility restriction (chair or platform scale); this tool also seems to compensate for the lack of malnutrition screening tools or, more generally, for the absence of written documents (protocols, guidelines) that provide advice for assessing body weight.

Moreover, the structural and process indicators are, at times, necessary but not sufficient to ensure quality: due to variability in the effective use of such tools and in the mode and frequency of documentation describing the care processes, these indicators could be insufficient. The outcome indicators are generally considered to be the best indicators of the quality of care because they summarise residents’ health status [2]. Conversely, interventions that aim to modify the structural or process indicators are generally simpler and less expensive than those that directly and specifically intervene at the outcome indicator level. In this sense, identifying the predictors of malnutrition risk (after controlling for individual factors) may be useful for prioritising interventions. Thus, though enforcing that NHs be provided with a proper scale would be an easy-to-perform and low-cost intervention, it needs to be accompanied by specific staff training aimed at increasing awareness towards malnutrition risk, as well as by instructions for taking anthropometric measurements and assessing malnutrition risk properly.

Our results also highlight the significant association between some individual factors and the risk of malnutrition, in concordance with the results from previous studies [9, 21]: physical disability, cognitive impairment, functional ability to eat, age, and the place where the residents ate lunch, all emerged as predictors of malnutrition risk, independently of the NH characteristics. In our opinion, the variable “eating in the bedroom”, which emerged as the one with the highest OR value, should be considered as a proxy for overall health status, including both a high level of disability (i.e. the subjects are bedridden) and the presence of acute diseases or exacerbations of chronic diseases that impede them from moving from the bedroom. In fact, having the meals in the dining room with the other residents is a practice that, with the help of healthcare staff, is generally encouraged, at least in the Tuscan NHs, also for elderly with disabilities. This consideration could explain the low weight of the Barthel Index in predicting the outcome (OR = 1.01).

The individual factors that had a role in predicting the outcome are difficult to modify and should probably suggest greater need for assistance in the nutrition care process, as well as a more frequent and in-depth assessment of malnutrition risk.

Our results confirm that when assessing the quality of the NHs, it is relevant to include risk adjustment models in the calculation of outcome indicators [46–49].

The dissemination of quality principles in NHs using specific training programmes, the implementation of monitoring systems, and the introduction of specific tools (i.e., the aims of the overall project in which the present study is included) are an answer to both the health needs of elderly residents in long-term care and the need for the most appropriate allocation of the available resources. Enhancing the quality of nutritional care might provide medium- and long-term benefits in residents’ health status and quality of life, ultimately reducing the costs related to the care process [18, 50].

The limitations of this study are primarily related to the mode of NHs enrolment that, being voluntary, might have produced a best-case scenario artefact, while limiting the generalisability of the results. Indeed, it can be easily assumed that only the NHs whose staff and directors were more eager to improve quality decided to join the project.

Another limitation is in the high number (89) of observers collecting the data, who were staff members of the NHs and not trained researchers. This might have limited the reliability of the information, although the observers did receive a specific training. This choice was made to allow the operators to be directly involved and responsible, to stimulate their awareness towards health and quality of care in NHs and to provide them with operational tools that could be used immediately, regardless of the research outcomes. In addition, this strategy allowed us to evaluate the applicability of the tools in the “real world”, so that this study combined research with a training opportunity offered to NHs staff, which learned how to assess and manage malnutrition risk through the correct routine detection of simple anthropometric measurements, as suggested by other authors [51–53]. Finally, the pre-existing therapeutic relationship between residents and staff might have facilitated data collection, which was challenged by the characteristics of the participants, who were frail elderly persons with comorbidities, disability and cognitive impairment.

A further limitation of the survey concerns the limited number of structural and process indicators that were investigated, especially those related to staffing (education, special training).

Finally, the study presents the typical limits of cross-sectional studies: further studies are required to assess the association between process, structural and outcome indicators in a longitudinal perspective. Moreover, since the nutritional status of the subjects may affect their physical functioning, another limitation is related to the possible association between nutritional status (i.e. our outcome measure) and the case-mix (i.e. our confounders), and this aspect is hard to deeply assess in cross-sectional studies.

## Conclusion

When its strengths and limitations are honestly balanced, we believe that this study provides an original contribution to the assessment of quality of NH, specifically in an important area such as malnutrition risk. Based on our findings, efforts should be made to improve the quality of the nutritional care provided, which is in general suboptimal and in some aspects definitively poor.

The availability of a scale to weigh residents with severely impaired mobility emerges as the unique structural or process indicator significantly associated with the outcome indicator. Therefore, equipping NHs with this simple instrument is a priority, possibly in combination with a specific training of the staff.

Finally, when assessing the quality of the NHs, it is relevant to develop risk adjustment models in the calculation of outcome indicators.

## Abbreviations

NH: Nursing home
MUST: Malnutrition universal screening tool
BMI: Body mass index BMI
MUAC: Middle upper arm circumference
KH: Knee height
UL: Ulna length
AIC: Akaike information criteria
ROC: Receiver operating characteristic
EBS: Eating behaviour scale
GDS: Global deterioration scale
SOSIA: Scheda di Osservazione Intermedia Assistenza
SVAMA: Scheda per la Valutazione Multidimensionale dell’Anziano
DC: Disease count
